# Can Phosphodiesterase 4 Inhibitor Therapy Be Used in Respiratory Diseases Other Than Chronic Obstructive Pulmonary Disease?

**DOI:** 10.7759/cureus.27132

**Published:** 2022-07-22

**Authors:** Mastiyage R Goonathilake, Sara Waqar, Sheeba George, Wilford Jean-Baptiste, Amina Yusuf Ali, Bithaiah Inyang, Feeba Sam Koshy, Kitty George, Prakar Poudel, Roopa Chalasani, Lubna Mohammed

**Affiliations:** 1 Research, California Institute of Behavioral Neurosciences & Psychology, Fairfield, USA; 2 Pediatrics, California Institute of Behavioral Neurosciences & Psychology, Fairfield, USA; 3 Internal Medicine, California Institute of Behavioral Neurosciences & Psychology, Fairfield, USA

**Keywords:** bronchiectasis, roflumilast, pde inhibitors, cystic fibrosis, chronic cough, asthma, pde4 inhibitors

## Abstract

Selective phosphodiesterase 4 (PDE4) inhibitors have been extensively studied for the treatment of various respiratory diseases due to their broad anti-inflammatory and/or bronchodilator effects. Roflumilast, an oral selective PDE4 inhibitor, is currently used as a second-line treatment in patients with chronic obstructive pulmonary disease (COPD) with chronic bronchitis. Despite its proven efficacy in other respiratory disorders, including asthma, no other PDE4 inhibitor is approved for respiratory pathologies. This systematic review summarizes the therapeutic action of PDE4 inhibitors, their limitations, recent therapeutic success, and future targets for their use in respiratory diseases other than COPD. An electronic literature search was conducted on four databases, namely, PubMed, PubMed Central, Google Scholar, and ScienceDirect, to collect data on related studies done in humans and published in the English language in the last five years. After extensive analysis and quality appraisal, 11 studies were eligible and thus included in this review, consisting of two randomized controlled trials (RCT), one systematic review and meta-analysis, and eight literature reviews. Roflumilast is not approved for the treatment of asthma due to associated adverse effects and comparable efficacy to inhaled corticosteroids, which are considered the mainstay of asthma maintenance therapy. Hence, the importance of balancing the efficacy with minimizing the side effects is highlighted. Tanimilast (CHF6001), an inhalational selective PDE4 inhibitor, and ensifentrine, a combined PDE3/4 inhibitor, demonstrate the recent therapeutic success in asthma and warrant further large-scale clinical studies. Future researchers will focus on the specific endotype than the phenotype in asthma as a meaningful therapeutic approach due to the high heterogeneity noted in asthma. Current evidence suggests the possibility of PDE4 inhibitors as a novel therapeutic option for chronic cough, allergic rhinitis, and cystic fibrosis. Further evidence from new studies is eagerly anticipated to better understand the efficacy and safety of PDE4 inhibitors in these respiratory diseases.

## Introduction and background

Lungs, the main organ in the respiratory system, expand and contract about a thousand times a day providing an efficient gas exchange [1]. Diseases or pathologies in the lungs and airways that develop slowly and worsen over time are called chronic respiratory diseases [1]. Chronic obstructive pulmonary disease (COPD), asthma, and occupational lung diseases are among the most common and major chronic respiratory diseases [2]. According to the Global Burden of Diseases, Injuries, and Risk Factors Study (GBD) 2017, chronic respiratory diseases are one of the leading causes of death and disability in the world, with the highest prevalence in high-income regions [3]. Nearly 545 million individuals (7.4% of the world’s population) live with chronic respiratory problems [3]. The economic burden associated with asthma, COPD, and other chronic respiratory disorders is among the highest compared to other chronic diseases [4].

According to the Global Initiative for Asthma, 2019, asthma is characterized as chronic airway inflammation and hyperresponsiveness with variable expiratory airflow limitation [3]. It is the most common non-communicable disease in children, and 40% of children and 50% of adults diagnosed with asthma have uncontrolled disease, representing an unmet medical need [3,4]. Despite differences in etiology, symptoms, and prognosis, asthma and COPD share the common pathogenesis of airway inflammation [5]. Medications that selectively target the disease pathology will increase the chances of therapeutic success [5].

Phosphodiesterase (PDE) is an enzyme involved in the pathogenesis of various chronic inflammatory diseases and degenerative diseases in humans [6]. PDE acts by hydrolyzing the intracellular nucleotide cyclic adenosine monophosphate (cAMP) and cyclic guanosine-5-monophosphate (cGMP) to its inactive compounds, adenosine-5-monophosphate (5-AMP) and guanosine-5-monophosphate (5-GMP), respectively [6]. PDE inhibitors are a major class of drugs currently being investigated as a treatment strategy for COPD, asthma, depression, cognitive and affective disorders, atopic dermatitis, and fragile X syndrome [7].

The selective phosphodiesterase 4 (PDE4) enzyme, which is encoded by four genes, is a member of the PDE superfamily of 11 subtypes [8]. As it is specifically found in inflammatory cells and airway smooth muscle cells, PDE4 inhibitors are considered a therapeutic target for inflammatory respiratory diseases such as COPD and asthma [8,9]. However, roflumilast is the only PDE4 inhibitor currently approved for the treatment of respiratory disorders and is used as a second-line medication for severe COPD with chronic bronchitis [6]. Other PDE4 inhibitors that have been approved include apremilast for psoriatic arthritis and plaque psoriasis and crisaborole for the topical treatment of atopic dermatitis [6].

Clinical data provide promising results for the potential use of PDE4 inhibitors in asthmatic patients [4]. Its effectiveness has also been tested for various other inflammatory respiratory diseases, including allergic rhinitis, non-cystic fibrosis bronchiectasis, and chronic cough [6,10]. However, PDE4 inhibitors are not approved as a treatment strategy for any other respiratory diseases except COPD [7]. This systematic review summarizes the recent evidence on PDE4 inhibitors concerning their therapeutic potential, limitations for approval as treatment options for respiratory disorders other than COPD, and the recent advances to overcome the limitations.

## Review

Methodology

The Preferred Reporting Items for Systematic Reviews and Meta-Analysis (PRISMA) guidelines 2020 were followed in this systematic review, and the population, intervention, and outcome with or without a control (PICO) format was included in this study pattern [11].

Inclusion and Exclusion Criteria

All studies related to the topic, published in the English language, across the globe within the last five years (2017-2022) where the free full-text article is available or can be received from the author were included. The study population considered was humans without limitation of age or sex who were affected by respiratory disorders other than COPD. The overall effect of the selective PDE4 inhibitor therapy (intervention) on the disease outcome compared to the conventional treatment or placebo (control) or without any comparison group was assessed. All types of study designs were included without any restrictions. All other articles published before 2017, non-English-language studies, animal studies, non-full-text articles, book articles, and gray literature were excluded from the study.

Information Sources and Search Strategy

A detailed search was done on four databases, namely, PubMed, PubMed Central, Google Scholar, and ScienceDirect, using the relevant keywords. Medical Subject Heading (MeSH) search blocks with Boolean operators were used in the PubMed database search, and appropriate filters were used according to the availability in the selected databases. The data were searched from all databases lastly on April 16, 2022. The search strategy including the relevant keywords and MeSH terms used is listed in Table 1.

**Table 1:** Search strategy for different databases and their search results. PDE4 inhibitors: phosphodiesterase 4 inhibitors; COPD: chronic obstructive pulmonary disease

Database	Search strategy	Filters	Search result
PubMed	Asthma OR Bronchiectasis OR chronic cough OR (((“Asthma/drug effects” [Majr] OR “Asthma/drug therapy” [Majr] OR “Asthma/prevention and control” [Majr] OR “Asthma/therapy” [Majr] )) OR (“Bronchiectasis/drug therapy” [Majr] OR “Bronchiectasis/prevention and control” [Majr] OR “Bronchiectasis/therapy” [Majr] )) AND Phosphodiesterase 4 inhibitors OR PDE inhibitors OR Roflumilast OR (“Phosphodiesterase 4 Inhibitors/administration and dosage” [Mesh] OR “Phosphodiesterase 4 Inhibitors/adverse effects” [Mesh] OR “Phosphodiesterase 4 Inhibitors/therapeutic use” [Mesh] OR “Phosphodiesterase 4 Inhibitors/toxicity” [Mesh])	Humans, English language, 2017–2022, free full text	268
PMC	Asthma OR Bronchiectasis OR Chronic cough AND PDE 4 inhibitors	Five years	355
Google Scholar	Asthma OR Bronchiectasis OR Chronic cough AND PDE 4 inhibitors	2017–2022, review articles	2,290 (First 500 records were identified)
ScienceDirect	PDE4 inhibitor therapy in Non-COPD respiratory diseases	2017–2022, review and research articles	107

Data Collection and Study Selection

Study selection was done by two researchers independently according to the inclusion and exclusion criteria. Full articles were analyzed extensively, and any discrepancies were reevaluated and reassessed by both researchers to reach common ground. Studies that were focused on our topic, fit our inclusion and exclusion criteria, and were of good quality were chosen for this study.

Results

**Figure 1:**
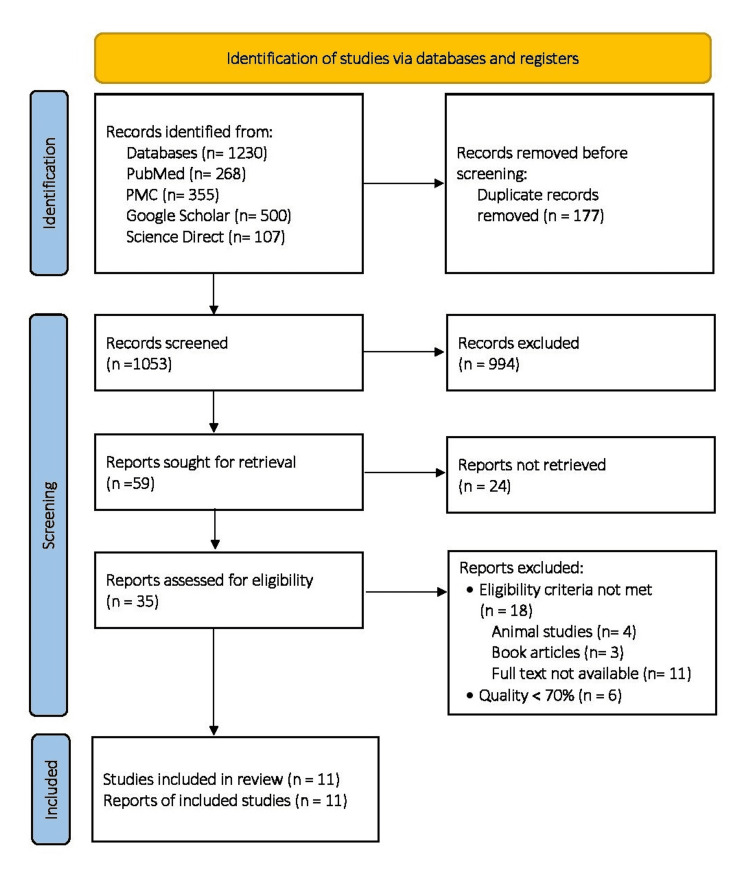
PRISMA 2020 flowchart of the databases and studies. PRISMA: Preferred Reporting Items for Systematic Reviews and Meta-Analyses

Among the identified 1,230 studies, 177 duplicates were removed using the EndNote X9 version and manually. The remaining 1,053 articles were initially screened based on the title and abstract. Among them, 994 studies were excluded as they were irrelevant to the study, and the remaining 59 studies were sought for retrieval for further screening. Only 35 studies remained for the assessment because 24 studies were not retrieved. Full articles of 35 studies were assessed extensively based on the eligibility criteria and quality. Finally, a total of 11 studies were included in this systematic review. Figure 1 depicts the search process used for this review in the form of a PRISMA flow diagram [11].

Quality Appraisal

The risk of bias in individual studies was reduced by assessing the quality by two independent researchers with the use of relevant quality assessment tools. Studies that had a quality above 70% or get an overall “Low Risk” in risk of bias were included while the studies that did not fit the criteria or that we found biased were excluded. The summary of the quality appraisal process of selected studies is included in Table 2.

**Table 2:** Quality Appraisal tools of final studies RCTs: randomized controlled trials; RoB: risk of bias

Quality assessment tool	Type of study	Total score	Accepted score (>70%)	Accepted studies
Assessment of Multiple Systematic Reviews (AMSTAR 2) [12]	Systematic review and Meta-analysis	16	12	Luo et al. (2018) [9]
Scale for the quality Assessment of Narrative Review Articles (SANRA 2) [13]	Narrative review	12	9	Chinn et al. (2020) [4] Facchinetti et al. (2021) [5] Kawamatawong et al. (2021) [6] Phillips et al. (2020) [8] Syfridiana et al. (2021) [14] Zuo et al. (2019) [15] Li et al. (2018) [16] Matera et al. (2021) [17]
Cochrane risk-of-bias tool (RoB 2) [18]	RCTs	7	5 (Low Risk)	Bjermer et al. (2019) [19] Juthong et al. (2022) [20]

Data Analysis

This systematic review describes the study results based on their outcomes, applicability, and limitations as a narrative synthesis because inter-variability was noted between the studies such as heterogeneity of study designs, participants, interventions, and outcome measures. All articles included in the study were reviewed and analyzed extensively and tabulated into (1) randomized controlled trials (RCT), (2) systematic review and meta-analyses, and (3) review articles. Table 3 summarizes the relevant results extracted from the selected studies.

**Table 3:** Summary of the selected studies. cAMP: cyclic adenosine monophosphate; PDE4: phosphodiesterase 4; COPD: chronic obstructive pulmonary disease; FEV1: forced expiratory volume in the first second; LAR: late asthmatic response; RCT: randomized controlled trial

Study source	Study name	Study type	Study objective	Related conclusion
Chinn et al. (2020) [4]	Cyclic AMP in dendritic cells: A novel potential target for disease‐modifying agents in asthma and other allergic disorders	Literature review	Review the role of dendritic cells and cAMP as potential disease-modifying therapies in asthma and other allergic disorders	Propose to design drugs that selectively raise cAMP in dendritic cells as a novel disease-modifying therapy for allergic asthma
Facchinetti et al. (2021) [5]	Tanimilast, a novel inhaled PDE4 inhibitor for the treatment of asthma and chronic obstructive pulmonary disease	Literature review	Review main preclinical and clinical studies conducted during the development of Tanimilast and identify subgroups of patients with possible therapeutic success	Tanimilast demonstrates good anti-inflammatory properties in both COPD and asthma. Phase IIa clinical studies used in asthma demonstrated significant LAR to inhaled allergens and numerical reduction in sputum eosinophilia
Kawamatawong et al. (2021) [6]	Phosphodiesterase-4 inhibitors for non-COPD respiratory diseases	Literature review	Review the evidence on the effectiveness of Roflumilast and other PDE4 inhibitors in chronic inflammatory respiratory diseases beyond COPD including certain COPD phenotypes with comorbidities	Roflumilast and selective PDE4 inhibitors have demonstrated a broad spectrum of anti-inflammatory effects on chronic respiratory diseases including asthma, asthma-COPD overlap syndrome, and COPD with comorbidities. Further well-designed clinical studies will be helpful
Phillips et al. (2020) [8]	Inhaled phosphodiesterase 4 (PDE4) inhibitors for inflammatory respiratory diseases	Literature review	Summarize the clinical structure, pharmacological, and clinical details of inhaled PDE4 inhibitors	CHF 6001 as the only inhaled PDE4 inhibitor currently advancing through clinical development has promising results with minimal systemic adverse effects in phase II clinical trials in asthma
Luo et al. (2018) [9]	Efficacy and safety of phosphodiesterase 4 inhibitors in patients with asthma: a systematic review and meta-analysis	Systematic review and meta-analysis	Evaluation of the effects of PDE4 inhibitors on clinical outcomes in patients with asthma	Oral PDE4 inhibitors improve lung function, asthma control, and asthma exacerbations with the expense of increased adverse events. Oral PDE4 inhibitors including roflumilast 500 µg may be an alternative treatment to regular bronchodilators and inhaled controllers in patients with mild asthma
Syfridiana et al. (2021) [14]	Roflumilast: review of phosphodiesterase-4 inhibitor as asthma therapy	Literature review	Determine the efficacy and safety of using roflumilast as a therapeutic option in asthmatic patients	Numerous clinical studies conducted on the effectiveness of roflumilast therapy in asthma (phase I-III) demonstrated significant improvement in FEV1. Statistically, a significant difference was not noted between the doses of 250 and 500 µg of roflumilast. Combination therapy with montelukast demonstrated comparative improvement in lung functions and respiratory symptoms
Zuo et al. (2019) [15]	Phosphodiesterases as therapeutic targets for respiratory diseases	Literature review	Discuss PDE subtypes and the role of selective PDE inhibitors in the therapeutic application for COPD and asthma	PDEs are an attractive pharmaceutical target for COPD and asthma treatment. Dual PDE4/3 inhibitor (RPL554) demonstrated anti-inflammatory and airway-modulatory effects in phase I clinical trials. Further clinical studies to explore the real pharmaceutical target of RPL554 were recommended
Li et al. (2018) [16]	Phosphodiesterase-4 inhibitors for the treatment of inflammatory diseases	Literature review	Summarize the chemical skeleton and pharmacological and clinical details of the licensed PDE4 inhibitors in the process	Various adverse effects associated with PDE4 inhibitors are the primary bottleneck in new drug development. Three possible strategies to avoid this problem were described
Matera et al. (2021) [17]	New avenues for phosphodiesterase inhibitors in asthma	Literature review	Discuss the progress made in recent years regarding PDE4 inhibitors in the treatment of asthma	No PDE inhibitor has yet reached the market as a therapeutic option for asthma. The current focus is on the development of PDE inhibitors that interact simultaneously with different PDE types. CHF6001 and RPL554 are the PDE4 inhibitors under development for asthma to date
Bjermer et al. (2019) [19]	Efficacy and safety of a first-in-class inhaled PDE3/4 inhibitor (Ensifentrine) vs Salbutamol in asthma	RCT	Investigate the dose-response and the pharmacology of a single dose of ensifentrine nebulizer suspension	Single-dose ensifentrine demonstrated dose-dependent bronchodilation which is effective as a therapeutic dose of nebulized salbutamol and did not show the systemic safety issues of β2 agonists
Juthong et al. (2022) [20]	Efficacy of roflumilast in bronchiectasis patients with frequent exacerbations	RCT	Assess the efficacy of roflumilast on the exacerbation of bronchiectasis	Roflumilast did not significantly affect the rate of exacerbation or the quality of life. Improvement in FEV1 was noted in the roflumilast group compared to the placebo group

Discussion

This section describes the PDE enzyme, therapeutic benefits of PDE inhibitors, recent advances in the development of selective PDE4 inhibitors, and current evidence and future targets of their use in various respiratory diseases beyond COPD.

Phosphodiesterase and cAMP

PDE enzyme in mammals is classified into 11 subfamilies based on kinetics, substrate selectivity, and their distribution in cells and tissues [21]. It modulates intracellular signal transduction by catalyzing the hydrolysis of cAMP and cGMP into their inactive metabolites 5-AMP and 5-GMP, respectively [5,6]. Identification of this enzyme about 60 years ago opened the gates to an important area of clinical research as inhibition of this enzyme provides an enormous potential for therapeutic benefit in many pathological conditions [22]. Current evidence supports that different PDE subtypes have their own characteristics such as PDE1, PDE2, PDE3, PDE10, and PDE11 degrade both cAMP and cGMP; PDE5, PDE6, and PDE9 only degrade cGMP; and PDE4, PDE7, and PDE8 degrade only cAMP [22]. PDE4 and PDE5 are the most important isoforms related to respiratory disease [21].

cAMP is an intracellular second messenger that is produced by the conversion of adenosine triphosphate (ATP) by the enzyme adenylyl cyclase (AC) after activation of G-protein coupled receptors (GPCR) [21,22]. cAMP plays a key role in cellular function and its signaling which is compartmentalized within cells explains the vast area of action sometimes even opposing effects [21]. PDE inhibitors that prevent hydrolysis of this unstable compound provide therapeutic benefits by increasing cellular cAMP levels [21].

PDE4 Inhibitors and Roflumilast

Genetic encoding and tissue distribution classify the PDE4 enzyme into four subtypes, namely, PDE4A, PDE4B, PDE4C, and PDE4D [6]. The genes encoding these subtypes also encode several different isoforms (three-eleven) within the subfamily [15,22]. Even though PDE4 are mostly abundant in inflammatory cells, airway cells, and lung tissues, they are also present throughout the body, including, but not limited to, the brain, heart, kidney, skeletal muscle, skin, testis, and liver [6,15]. Evidence support that PDE4 isoform expression in lung tissue varies depending on their clinical status such as in patients with COPD and asthma compared to healthy individuals [15]. Regulation of fundamental functions is the key role of selective PDE4 inhibitors, which comprises stabilization of endothelial and epithelial barriers, modulation of the inflammatory response, and cognitive and/or mood function [22].

**Figure 2:**
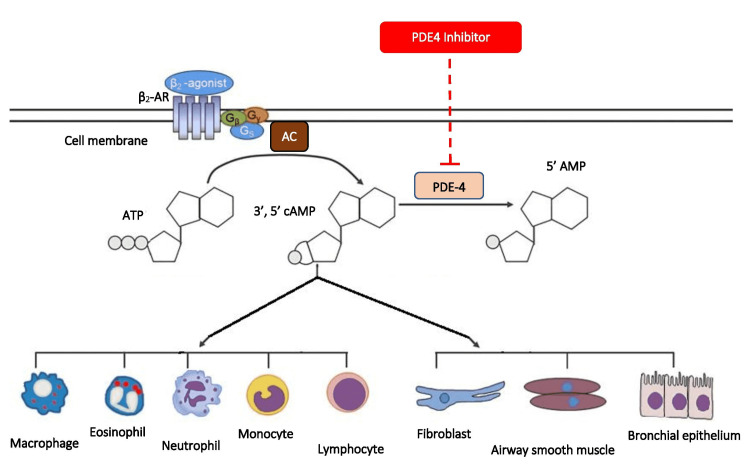
Mechanism of action of selective PDE4 inhibitors. β_2_-AR: β_2_ agonist receptor; AC: adenylyl cyclase; ATP: adenosine triphosphate; cAMP; cyclic adenosine monophosphate; 5'AMP: adenosine-5-monophosphate; PDE 4: phosphodiesterase 4 Authors’ own creation modified according to [15] and [22].

Roflumilast is the most extensively studied second-generation PDE4 inhibitor for respiratory diseases and is the only approved PDE4 inhibitor for respiratory pathology, i.e., COPD [6,8]. Compared to the non-selective PDE inhibitor theophylline, both roflumilast and its metabolite roflumilast-N-oxide are potent selective PDE4 inhibitors that act on inflammatory cells and the structural cells of the respiratory system involved in the pathogenesis of chronic respiratory diseases [6]. The pathophysiological basis of preventing inflammation by roflumilast has been studied extensively. Roflumilast acts on the lung macrophages inhibiting inflammatory cytokine release, eosinophils inhibiting reactive oxygen species formation (ROS), and neutrophils suppressing the release of their inflammatory mediators [6]. They also act on the airway smooth muscle cells and produce an inhibitory effect on contractile activity promoting bronchodilation [6]. A synergistic effect of dexamethasone on airway smooth muscle cells, when given in combination with formoterol, a long-acting β_2_ agonist (LABA), was also noted [6,23]. Some other effects of roflumilast on respiratory pathologies include (1) inhibition of profibrotic growth factor (TGF-β), (2) attenuation of fibroblast chemotaxis that promotes airway and lung fibrosis, (3) activation of cystic fibrosis transmembrane conductance regulator (CFTR) in airway epithelial cells, (4) inhibition of release of tumor necrosis factor-α (TNFα) by bronchial epithelial cells, and (5) decrease in the expression of MUC5AC (predominant mucin gene expressed in healthy airways and overexpressed in asthmatic and COPD patients) in human airway epithelial cells [6]. It also exerts favorable effects on the cigarette smoke-injured human bronchial epithelium by improving ciliary motility and increasing airway surface liquid (ASL) hydration, facilitating mucus dehydration and mucus clearance in COPD with chronic bronchitis and other suppurative airway diseases [6]. Figure 2 describes the mechanism of action of PDE4 inhibitors.

Despite its high potential to provide therapeutic benefit in many disease pathologies, only three PDE4 inhibitor drugs (roflumilast, crisaborole, apremilast) are currently being approved [8]. The main reasons identified for this delay in therapeutic success are the narrow therapeutic index and the intolerable adverse effect profile [8]. The most commonly observed adverse effects are nausea, diarrhea, abdominal pain, loss of appetite, weight loss, headache, and sleep disturbances [10]. Also, studies on the efficacy of roflumilast revealed that the maximum tolerated dose is near the bottom of the efficacy dose-response curve [8]. These have prompted current research to consider developing novel PDE4 inhibitors which enhance treatment efficacy and avoid adverse effects [7].

PDE4 Inhibitors in Asthma

Inhaled corticosteroids (ICS) and LABA are the mainstay of maintenance therapy in asthma. Despite their therapeutic efficacy, a considerable proportion of asthmatic patients still experience recurrent symptoms [24]. The epidemiological data on uncontrolled asthma and the economic burden per each asthma patient per year, which has been calculated as USD 1,000, highlight the necessity of novel therapeutic options in asthma management [22]. Even though researchers and clinicians repeatedly expressed the possibility of using PDE inhibitors in asthma therapy due to their effective bronchodilator and anti-inflammatory properties, none of the PDE4 inhibitors has entered the market as asthma therapy in the past three decades [17].

Studies on roflumilast demonstrated a reduction in late asthmatic response (LAR) and prevention of subsequent increase in bronchial reactivity following an allergen challenge. But a considerable effect on the acute-phase response (bronchoconstriction) was not demonstrated [25,26]. Further studies demonstrated improvement in lung functions of asthmatic patients when combined with ICS or montelukast [27,28]. Because ICS has a flat dose-response in airway caliber to high doses, adding on drugs such as LABA or montelukast is preferred over increasing the ICS dose and add-on LABA is shown to be more effective than add-on montelukast [6]. A short-term study done by adding roflumilast 500 µg and montelukast 10 mg to ICS/LABA compared to adding only montelukast 10 mg to ICS/LABA in patients with poorly controlled asthma showed comparative improvement in forced expiratory volume in the first second (FEV1) in the roflumilast group [14,27]. This potential benefit of improving lung function on FEV1 and forced vital capacity (FVC) by adding roflumilast can be explained as attenuation of airway inflammation for ICS and/or synergistic bronchodilator effect with LABA [6]. The mechanism of PDE4 inhibitors and LABA improving the clinical efficacy of glucocorticoids in inflammatory lung diseases has been explained as an interplay between the glucocorticoid receptor and the cAMP receptor pathway [29]. However, a paucity of further clinical studies on roflumilast in asthma therapy was noted due to its comparable efficacy to ICS with the expense of numerous adverse effects [6]. Further studies done in this field have identified three main strategies as effective to overcome these barriers. They are designing potent isoform-specific inhibitors or allosteric modulators, changing the route of administration by designing inhalational preparations and combining therapy with other medications [16]. Table 4 summarizes the details of other important PDE4 inhibitors studied in asthma.

**Table 4:** Important PDE4 inhibitors studied in asthma. ICS: inhaled corticosteroid; EAR: early asthmatic response; LAR: late asthmatic response

Author and year	Patient characteristics	Intervention	Duration	Out come	Comment
Singh et al. (2010) [30]	Atopic asthma-ICS naive	Inhaled isoform-specific PDE4B (GSK 256066) 87.5 µg versus placebo	Seven days	Demonstrated significant protective effects on both EAR and LAR to allergen challenge.	No longer in the development process due to its poor pharmacokinetic properties.
Singh et al. (2016) [31]	Atopic asthma-ICS naive	Inhaled CHF6001 400 µg/1,200 µg vs placebo OD via DPI	Nine days	Demonstrated significant attenuation of LAR to allergen challenge- Non-significant reduction in sputum eosinophil count was noted	Promising results warrant further research
Leaker et al. (2014) [32]	Atopic asthma-ICS naive	Oral MEM1414 600 mg BID vs placebo	Two weeks	Demonstrated significant reduction of LAR to allergen challenge. No effect was noted on EAR	Associated side effects abandoned further research
Bjermer et al. (2019) [19]	Asthma	Nebulized ensifentrine 0.4, 1.5, 6, and 24mg vs salbutamol 2.5 and 7.5 µg vs placebo		Demonstrated significant dose-dependent bronchodilation compared to placebo. Efficacy was comparable to the therapeutic dose of nebulized salbutamol with good tolerability. Did not show β2 agonist-associated systemic adverse effects	Promising results warrant further research

GSK256066, an inhalational isoform-specific PDE4B inhibitor, has demonstrated protective effects on both early asthmatic response (EAR) and LAR to inhaled allergens [8,30]. However, further studies on this drug were prevented due to its poor chemical properties which makes it difficult to exert a good pharmacological effect [8,30]. Tanimilast (CHF6001), an inhaled selective PDE4 inhibitor that is about seven times more potent compared to roflumilast, has demonstrated significant inhibition of allergen-induced LAR in atopic asthmatics with minimal adverse effects in phase II clinical trials [5,31]. This has reached phase III clinical development in COPD patients with good tolerability, safety profile, and no evidence of class-related adverse effects [5]. Another study on Tanimilast describes that it has a greater effect on Th1 cytokines compared to corticosteroids. This suggests its potential role in the management of severe asthma [33].

Dual PDE inhibitors were developed to achieve optimal anti-inflammatory and bronchodilator action at a concentration that does not cause unwanted adverse effects [17]. Ensifentrine (RPL554) dual PDE 3/4 inhibitor has effective bronchodilator properties compared to a therapeutic dose of nebulized salbutamol and good tolerability without any β_2_ agonist systemic safety issues associated with salbutamol [15,19]. Promising results shown in this first RCT deemed the necessity of further research to confirm the effect of this new medication.

Novel Therapeutic Targets of PDE4 Inhibitors in Asthma

Selectively raising cAMP in the dendritic cells is a proposed novel therapeutic approach for allergic asthma. This follows the concept of focusing treatment on a specific endotype of disease (a distinct molecular mechanism) rather than the phenotype (disease characteristics independent of the mechanism) [4]. Asthma endotypes can be classified according to the predominantly involved cellular inflammatory mediators (eosinophils, neutrophils, mixed granulocytic) or type 2 helper T cell (Th2) high (allergic asthma) or non-type 2 (Th2 low) asthma [4]. Dendritic cells, which play an important role in inducing Th2 differentiation, play a key role in allergic asthma. Therefore, selectively inhibiting the PDE4 enzyme in dendritic cells will provide endotype-specific therapy for allergic asthma [4].

The importance of exploring the PDEs that were not fully investigated in the past for their ability to induce bronchodilation is another new suggestion in the research world [17,34]. Evidence supports the ability of PDE8 and PDE9 to induce bronchial smooth muscle relaxation in animals [34]. Potentially druggable targets in inhibiting these PDEs, which simultaneously interact with other PDEs, create new opportunities for future researchers and will be a more fruitful approach for improving the care of asthmatic patients [17,34].

Multiple therapy fixed-dose combination inhalers that contain dual PDE inhibitors and hybrid molecules with other bronchodilators are considered an effective therapeutic option for asthma as they can provide three to four complimentary effects together [17]. Scientists have also considered the development of hybrid molecules specifically designed to have multi-functional ligands containing two or more pharmacophores [17]. Future research focusing on these advanced methods will bring new hope for physicians caring for asthmatic patients.

PDE4 Inhibitors in Allergic Rhinitis

Allergic rhinitis (AR) is an inflammatory disorder of the nasal epithelium which occurs due to allergen exposure. About 15-38% of patients with allergic rhinitis are diagnosed with asthma, and 6-85% of patients with asthma get nasal symptoms [10]. Even though the combination of oral/intranasal antihistamines and intranasal glucocorticoids is considered the mainstay of therapy, the recently described non-Th2-mediated inflammatory pathway of AR does not respond well to the current treatment [35]. The efficacy of roflumilast in the treatment of AR was studied once and revealed that oral roflumilast was effective as an anti-allergy therapy but was associated with significant adverse effects [10]. Further research on developing topical PDE4 inhibitors acting directly on the nasal mucosa is considered an effective future approach to minimize the associated adverse effects [10,35].

PDE4 Inhibitors in Bronchiectasis

Bronchiectasis is a chronic suppurative respiratory disease characterized by abnormal bronchial dilatation, chronic productive cough, and recurrent infective exacerbations [21]. Chronic neutrophilic airway inflammation is a key component of pathogenesis leading to persistent bronchial dilation and lung damage [20]. Some studies have attempted to identify the potential role of PDE4 inhibitors in the management of bronchiectasis because of their possibility to modulate neutrophil function, improve mucus and ciliary function, and the bronchodilator effect [6,20]. A phase II clinical trial using roflumilast in symptomatic bronchiectasis patients demonstrated improvement in health-related quality of life measured by the COPD assessment test score and the St. George’s Respiratory Questionnaire (SGRQ) but the findings were not statistically significant [20]. The first RCT done to identify the efficacy of roflumilast in bronchiectasis concluded that roflumilast did not significantly affect the rate of exacerbations or quality of life. However, there was an improvement in lung function (FEV1) compared to the placebo group [20]. Therefore, further research including long-term prospective clinical studies using more bronchiectasis patients will be helpful to fill the clinical gap identified in this group of patients.

PDE4 Inhibitors in Chronic Cough

Chronic cough, as a troublesome complaint of a significant proportion of the population, may occur due to many clinical pathologies [21]. Even though many anti-tussive medications are on the market, the limitation of effective therapeutic strategies may have contributed to its high burden [21]. The lack of mechanistic research to elucidate the cough mechanism was identified as a key issue in this field [21]. Transient receptor potential (TRP) ion channels are associated with a chronic cough in several diseases [36]. TRP ion channels modulate inflammation, smooth muscle tone, and sensory afferent activation in the airways, and they get activated by chemical stimuli, temperature changes, mechanical stress, and osmotic stress [36]. PDE inhibitors, which have anti-inflammatory and bronchodilator properties, also cause suppression of TRP channels [37]. Therefore, the use of PDE inhibitors in the management of chronic cough was proposed to be effective, and among them, selective PDE3, PDE4, and PDE5 inhibitors have demonstrated the most significant cough-suppressive effects [37]. Further clinical studies in this field will hopefully lead to new effective therapies for chronic cough.

PDE4 Inhibitors in Cystic Fibrosis

Cystic fibrosis (CF) is an autosomal recessive lethal genetic disorder caused by mutations in the CFTR gene [38]. CFTR anion channel regulates ion and water transport across multiple epithelia, and impairment of its function in respiratory epithelia disrupts airway innate defense mechanisms resulting in bacterial colonization, excessive inflammation, and tissue damage in the respiratory system [38,39]. In research data, roflumilast has been shown to activate CFTR ion channels in the respiratory epithelium of normal human cells [6]. The previous conclusion that PDE inhibitors are ineffective in restoring CFTR-dependent ion transport in cystic fibrosis mutated cells was challenged by a recent study that demonstrated selective PDE4 inhibitor-associated amplification in the CFTR correctors and/or CFTR potentiators [38]. The first evidence that PDE4 inhibition causes NETosis in cystic fibrosis was provided by recent in vivo and in vitro studies in CF-relevant models [40]. The pathogenic role of neutrophil-derived free DNA, which is released in the form of extracellular traps (NETs), causing impaired lung function in CF, was the target mechanism in this study, and PDE4 inhibitors demonstrated significant control of NETosis of neutrophils migrated into the lungs [40]. These recent clinical advances provide a platform for future researchers to design further studies on the effectiveness of PDE4 inhibitors in CF, which has been a troublesome clinical entity cared for by pediatricians.

Limitations

The search strategy for this systematic review was limited to four databases where only papers published in the English language in the last five years (2017-2022) were included. This study merely analyzed free full-text studies and thus may have precluded the inclusion of important studies. Therefore, a data gap within the study area is a possibility. While the number and quality of included studies were adequate, the majority of them were narrative reviews. A paucity of prospective studies that could be useful in determining the genuine relationship of PDE4 inhibitor therapy in other respiratory diseases such as CF, chronic cough, bronchiectasis, and allergic rhinitis was noted.

## Conclusions

PDE inhibitors have been investigated as a treatment strategy for many disease pathologies in humans. Selective PDE4 inhibitors, which act by increasing cellular cAMP levels, have demonstrated their therapeutic potential in treating inflammatory respiratory diseases. Roflumilast, which is approved as second-line therapy in patients with COPD with chronic bronchitis, has also provided promising results in the management of asthma. Comparable efficacy to ICS with the expense of numerous adverse effects has been identified as the potential barrier to their use in clinical practice. Recent research aims to develop new PDE4 inhibitors with increased treatment efficacy and limited adverse effect profile. Novel isoform-specific selective PDE4 inhibitors, inhalational PDE4 preparations, and combined PDE inhibitors are being studied recently to overcome the problems and improve therapeutic benefits. Tanimilast (CHF6001), a more potent inhaled selective PDE4 inhibitor, and ensifentrine (RPL554), a PDE3/4 inhibitor, are two important drugs under research for asthma that have provided promising results. With the use of advanced technology, evidence supports the strong possibility of developing PDE4 inhibitors for the management of asthma in the future. More attention to the efficacy of PDE4 inhibitor therapy in chronic cough, CF, and AR will be a fruitful effort that could yield new therapeutics for the future.
